# Unravelling electro-chemo-mechanical interplay in layered oxide cathode degradation in solid-state batteries

**DOI:** 10.1126/sciadv.ady7189

**Published:** 2025-10-08

**Authors:** Xueli Zheng, Zhichen Xue, Hongchang Hao, Yukio Cho, Yuanshun Li, Chanho Kim, Pawel Czaja, Samuel Sanghyun Lee, Sharon Bone, Eleanor Spielman-Sun, Zhelong Jiang, X. Wendy Gu, Johanna Nelson Weker, Guang Yang, Jagjit Nanda

**Affiliations:** ^1^ Applied Energy Division, SLAC National Accelerator Laboratory, Menlo Park, CA 94025, USA.; ^2^ Department of Materials Science and Engineering, Stanford University, Stanford, CA 94305, USA.; ^3^ Department of Chemical Engineering, Stanford University, Stanford, CA 94305, USA.; ^4^ Chemical Sciences Division, Oak Ridge National Laboratory, Oak Ridge, TN 37830, USA.; ^5^ Department of Chemical and Biomolecular Engineering, University of Tennessee Knoxville, Knoxville, Tennessee 37996, USA.; ^6^ Institute of Metallurgy and Materials Science, Polish Academy of Sciences, 25 Reymonta St., 30-059 Krakow, Poland.; ^7^ Department of Mechanical Engineering, Stanford University, Stanford, CA 94305, USA.; ^8^ Stanford Synchrotron Radiation Lightsource, SLAC National Accelerator Laboratory, Menlo Park, CA 95014, USA.; ^9^ Institute of Bio- and Geosciences, IBG-3: Agrosphere Forschungszentrum Jülich, 52428 Jülich, Germany.

## Abstract

Solid-state batteries (SSBs) hold notable promise for advancing energy storage technologies. However, their commercial viability is limited by the poor cycle stability and complex degradation mechanism. This study underscores the pivotal role of electro-chemo-mechanical interactions in driving the failure of SSBs. Leveraging advanced x-ray imaging and spectroscopy techniques, we analyzed LiNi_0.8_Mn_0.1_Co_0.1_O_2_ (NMC811) cathodes from cycled Li*_x_*In||Li_6_PS_5_Cl (LPSC)||NMC811 SSBs, uncovering the interplay between microstructure, chemical heterogeneity, mechanical characteristics, and electrochemical performance. Our results show that revealing electro-chemo-mechanical interactions is essential to develop strategies to suppress the degradation of SSBs. Particularly, we revisit a LiNbO_3_ (LNO) coating layer to mitigate electrochemical degradation. The LNO@NMC811 cathode retains 116 milliampere-hours per gram after 200 cycles, showing excellent stability, while the uncoated NMC811 cathode keeps degrading over time, with suppressed chemical heterogeneity and mechanical failure. This work highlights the importance of synergizing advanced material design with coating techniques, ensuring uniform lithium flux and improving mechanical properties to achieve stable, high-performance SSBs.

## Teaser

Electro-chemo-mechanical couplings govern cathode degradation in solid-state batteries, while coatings effectively mitigate it.

## INTRODUCTION

High energy density and safety are key to energy storage technologies, particularly in portable electronics and electric vehicles (*1*). While lithium-ion batteries with nonaqueous liquid electrolytes offer the potential for further energy density improvements, safety concerns—primarily due to flammable electrolytes—along with the need for safe lithium metal anodes have driven the development of solid-state batteries (SSBs). SSBs, which use solid electrolytes, offer substantial advantages in addressing safety issues while enhancing both energy and power densities (*2*, *3*). However, the practical application and commercialization of SSBs face substantial hurdles. In addition to challenges like lithium dendrite formation, the degradation of cathode active materials limits the electrochemical performance of SSBs and poses a critical barrier to their widespread use (*4*, *5*).

Among all cathodes, layered oxide cathodes such as LiNi_1-x-y_Mn*_x_*Co_y_O_2_ (NMC) are regarded as some of the most promising candidates (*6*–*8*). Their robust crystal structures, combined with benefits such as high specific capacity and voltage, position them as ideal materials for advanced battery technologies (*9*). However, despite these advantages, the performance of NMC in sulfide-based SSBs falls notably short of its performance in conventional liquid electrolyte–based systems (*10*, *11*). NMC materials are commonly synthesized through a simple and cost-effective coprecipitation method followed by solid-state sintering, yielding spherical secondary particles composed of densely packed submicron primary grains (*12*, *13*). In liquid electrolyte–based lithium-ion batteries using NMC cathodes, the repeated insertion and extraction of lithium ions induce anisotropic expansion and contraction of the crystal lattice in NMC cathodes, generating internal stresses (*14*, *15*). Over time, these stresses accumulate and are relieved through intergranular fractures, such as microcracks, which propagate along grain boundaries during electrochemical cycling (*16*–*18*). Intergranular fractures reduce the electrical connectivity of active materials, leading to increased resistance. The newly exposed surfaces within the cracks react with the electrolyte, resulting in side reactions such as electrolyte decomposition and transition metal dissolution (*19*, *20*). These effects collectively accelerate capacity degradation and impedance growth. In liquid electrolytes, the ability of the electrolyte to penetrate cracks enables lithium-ion transport through newly formed active interfaces, preserving ionic diffusion pathways. However, solid electrolytes lack this capability, potentially leading to isolated domains within fractured particles where lithium diffusion is severely hindered (*21*, *22*). Systematically exploring how particle fracture accelerates the degradation of SSBs is essential to achieving high-performance SSBs with layered oxide cathodes.

Now, the main understanding of the failure mechanism of NMC in SSBs is that intense side reactions at the interface with sulfide-based electrolytes cause thick cathode layers and severe surface reconstruction, leading to capacity loss (*23*–*25*). Although chemical-mechanical simulations using cohesive zone and phase-field models have improved the understanding of the micro-mechanical fracture behavior in electrodes, the coupled electro-chemical-mechanical interplay mechanisms under real operating conditions remain insufficiently explored (*26*–*33*). Using state-of-the-art techniques to better understand the degradation mechanism of SSBs from the morphological and chemical dynamics is necessary.

In this study, we used advanced synchrotron x-ray microscopy and spectroscopy to investigate capacity loss in LiNi_0.8_Mn_0.1_Co_0.1_O_2_ (NMC811) cathodes using the sulfide solid electrolyte Li_6_PS_5_Cl (LPSCl). Specifically, we revealed substantial morphological changes in the NMC811 cathode after cycling, including extensive fragmentation and crack formation. The x-ray absorption spectra indicated spatial heterogeneity of nickel (Ni) oxidation states in the state of charge (SOC) across particles, suggesting variations in charge distribution and phase transitions. Cracks were found to exacerbate the interaction between the surface and bulk phases, further influencing the electrochemical performance of SSBs. Chemical heterogeneity, coupled with the crack distribution, demonstrates that poor electrolyte contacts and side reactions contribute to nonuniform SOC and uneven lithium-ion diffusion, leading to an electro-chemo-mechanical interplay that accelerates cathode degradation. To mitigate the degradation, we coated NMC811 with lithium niobate oxide (LiNbO_3_, LNO). Li*_x_*In||LPSCl||LNO@NMC811 full cells start with an initial capacity of 133 mAh/g and retain 116 mAh/g after 200 cycles, substantially outperforming the uncoated NMC811 cathode, which drops from 110 to 6 mAh/g. In addition, sulfur K-edge x-ray absorption spectroscopy showed that the LNO coating helped reduce side reactions at the cathode-electrolyte interface and minimized mechanical degradation, stabilizing the cathode’s chemical and mechanical integrity. Our results highlight how surface coatings like LNO can mitigate degradation mechanisms, enhance surface stability, and pave the way for developing safer and more efficient SSBs for energy storage applications.

## RESULTS

### Mechanical failure of NMC811 cathodes in SSBs

**Fig. 1.**
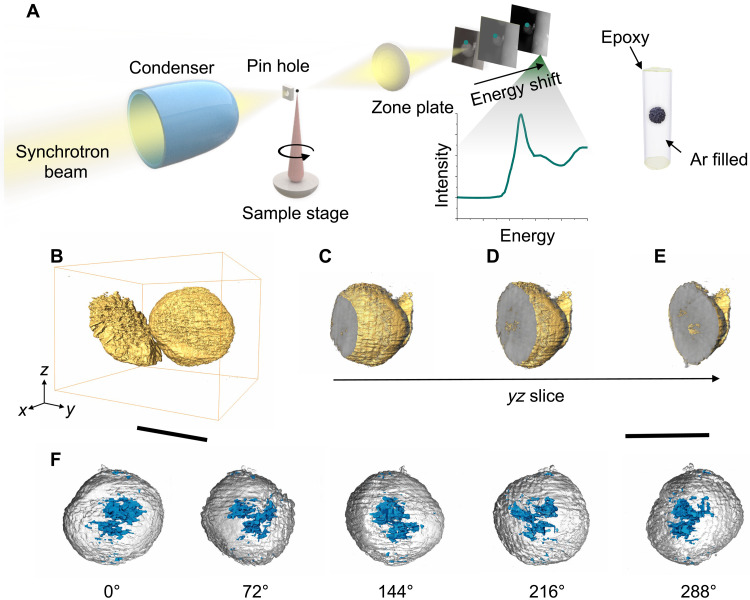
Mechanical failure of the cycled NMC811 cathode in SSBs. (**A**) Schematic diagram of the full-field TXM experimental setup and sample sealing environment. (**B** to **E**) 3D rendering of the cycled NMC811 cathode from a discharged cell obtained through computed tomography (B), with a slice through the particles along the *yz* direction [(C) to (E)]. (**F**) Internal cracks distribution rendering within the particles from different angles. [(C) to (F)] shares the scale bar. Scale bars, 10 μm [(B) and (C) to (F)].

We investigated the electrochemical failure and capacity loss in NMC811 cathodes from cycled discharged LPSCl SSBs using full-field transmission x-ray microscopy (TXM) at the Stanford Synchrotron Radiation Lightsource (SSRL) Beamline 6-2c. The experimental setup is depicted in Fig. 1A. Using a Fresnel zone plate as the objective lens, full-field images were captured with a spatial resolution of approximately 35 nm. The sample was rotated to image the selected particle from different angles, with measurements conducted at a range of incident energies around the Ni K-edge. The intensity evolution as a function of the incident energy at each pixel was recorded, generating an absorption spectrum for each, which was then used to construct a high-dimensional dataset. These data were analyzed using the in-house developed software TXM-Wizard (*34*). To prevent potential reactions between the air-sensitive LPSCl electrolyte and the NMC811 cathode surface, the sample was carefully prepared and sealed within a capillary filled with Ar for the experiment. Figure 1B captures images of two NMC811 particles collected at the discharged state after one formation cycle at C/20 and five additional cycles at C/10. The left particle has fractured into distinct segments, exhibiting substantial surface irregularities. Although the right particle appears largely spherical and intact from the outside, internal cracks are present. Even in the *yz* slice of this seemingly intact particle, substantial cracks are visible (Fig. 1, C to E). In Fig. 1F, the rendered cracks reveal a pattern where the fractures are concentrated in the core of the particle but are not fully interconnected. In the case of SSBs, these mechanical failures substantially affect ion diffusion within the NMC811 cathode, resulting in active material loss and capacity decay.

### Chemical variation of cycled NMC811 cathodes in SSBs

**Fig. 2.**
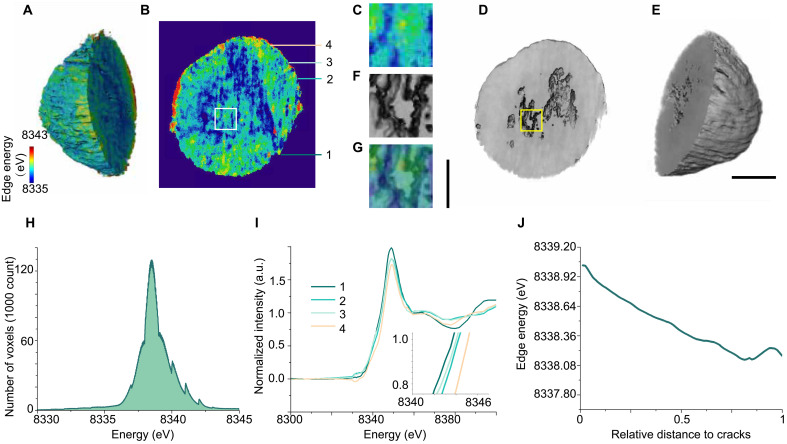
Chemical heterogeneity analysis of the cycled NMC811 cathode in SSBs. (**A** to **C**) 3D tomography of the imaged particle in (A) with a corresponding slice shown in (B) and a further zoomed-in view of the magnified area in (C) revealing detailed structural features. (**D** to **F**) 3D absorption spectrum imaging of Ni K-edge energy for the imaged particle, with rendering of the Ni K-edge energy map in (E), a slice through the particle showing Ni K-edge energy distribution in (D), and a magnified view of the slice in (F) highlighting the local variations in Ni oxide state. (**G**) The overlay of (C) and (F). (**H**) Ni K-edge energy histogram of the imaged particle. (**I**) Ni K-edge absorption spectra for several randomly selected surface pixels of the imaged particle. (**J**) Statistical profile of Ni-edge energy as a function of distance from a crack, where a distance of 0 denotes the point closest to the crack and a distance of 1 denotes the point furthest from the crack. Scale bars, 3 μm [(A), (B), (D), and (E)] and 1 μm [(D), (F), and (G)]. a.u., arbitrary units.

The formation of cracks prompted us to investigate the distribution of capacity loss within the particle by examining the spatial SOC variations. To achieve this, we conducted an analysis on a cycled and discharged NMC811 particle, obtained after one formation cycle at 0.05 C followed by five cycles at 0.1 C, focusing on how the internal SOC varied spatially within the damaged structure. By aligning multiple three-dimensional (3D) tomography datasets obtained at different energies, we constructed a comprehensive dataset of absorption spectra for all voxels. This provided insight into the spatial distribution of Ni oxidation states, which directly correlates with the internal SOC variations of the particle in the NMC811 cathode (*35*). As shown in Fig. 2 (A to C), clear heterogeneity in SOC is observed within the particle. Specifically, high-valent Ni outliers are predominantly found on the surface, while the bulk phase consists of segregated domains with relatively lower valence states. By examining the 2D slices of the 3D rendered tomography images (Fig. 2, D to F), it is evident that there are notable internal cracks within the particle and lead to isolated domains. The overlay of images in Fig. 2G shows a strong correlation between the SOC distribution pattern and the material’s morphological features. The isolated domain shown in Fig. 2F exhibits substantially higher SOC (Fig. 2C), which suggests hindered lithium-ion transport and localized capacity loss. Since the material originates from a discharged cell, the elevated SOC in these regions likely reflects areas that have become electrochemically inactive, directly contributing to the overall capacity degradation of the cathode. This heterogeneity in SOC can be directly linked to the loss of capacity. Higher valence states, such as those found in these isolated domains, represent lower electrochemical activity due to the reduced ability of Ni to participate in lithium-ion intercalation.

To further demonstrate the SOC heterogeneity, we plotted the Ni-K edge energy histogram in Fig. 2H and revealed a non-Gaussian peak distribution, further confirming the existence of varying valence states across the particle. When we examined the absorption spectra from several selected surface points, shown in Fig. 2I, we found notable differences in the spectra, indicating heterogeneous valence states across the surface. This surface Ni oxide state heterogeneity is potentially driven by the uneven attachment of the LPSCl electrolyte to the NMC811 cathode or by undesired surface side reactions occurring on the contact surface between the electrolyte and the cathode, which impede lithium-ion transport (*36*). These factors disrupt uniform ion transport and increase resistance at the particle-electrolyte interface, ultimately contributing to capacity loss. Moreover, the statistical profile of Ni K-edge edge energy as a function of distance from a crack, presented in Fig. 2J, shows a clear trend: Regions further from cracks exhibit lower SOC and higher electrochemical activity. Surface reactions do play a role in the observed heterogeneity, and the depth profile can be found in fig. S1; however, the disrupted connectivity within the particle due to cracking has a notable impact. In summary, cracks play a critical role in capacity loss by causing SOC heterogeneity, hindering lithium-ion diffusion, and accelerating electrochemical degradation within the NMC811 cathode. While in liquid electrolyte systems, electrolyte infiltration into the cracks can partially alleviate ionic transport limitations, in solid-state systems this effect is absent, making the impact of disrupted connectivity due to cracking even more pronounced.

### Electro-chemo-mechanical interplay in SSBs

**Fig. 3.**
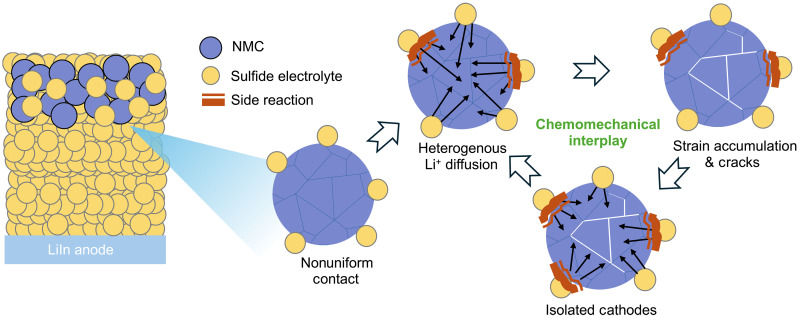
Schematic illustrating the electro-chemo-mechanical interplay responsible for NMC811 cathode degradation in SSBs.

Overall, in SSBs, the interaction between mechanical and chemical degradation mechanisms plays a crucial and often underappreciated role in the overall capacity loss. As shown in Fig. 3, in the case of NMC811 cathodes, the point-to-point contact between the solid electrolyte and the cathode particles leads to limited and heterogeneous lithium-ion diffusion paths (*37*). As the battery cycles, mechanical stresses from anisotropic grain level expansion and contraction of the cathode particles result in crack formation (*38*). In liquid electrolyte systems, these newly exposed interfaces enable the electrolyte to infiltrate the cracks, sustaining lithium-ion transport through the new pathways, albeit at the cost of forming a new cathode electrolyte interphase due to side reactions. However, in solid-state configurations, the inability of the solid electrolyte to penetrate these cracks results in a starkly different scenario. As a result, portions of the cathode material become “isolated” and unable to participate in the electrochemical processes. These isolated domains are regions where lithium-ion cannot diffuse, which causes the loss of activity and a gradual decrease in the overall performance of the cathode (*39*). Moreover, cracks and isolated domains induce a nonuniform SOC across the particle, which may lead to the accumulation of mechanical stress and further promote crack propagation during cycling. This creates a vicious cycle: Cracks impede lithium diffusion, which in turn increases mechanical stresses and promotes further crack formation. This electro-chemo-mechanical interplay, the combination of mechanical damage and restricted lithium-ion diffusion, ultimately accelerates capacity loss and impedance rise in SSBs. Addressing this interaction degradation is key to improving the stability and performance of SSBs.

### LNO coating enhanced SSB performance

Building on our findings, minimizing cracks and isolated domains in cathodes under operating conditions is critical to enhancing the cycling stability of SSBs. For sulfide-based SSBs, suppressing the surface side reactions between sulfides and NMC811 cathodes is also important. The highly reactive surfaces of NMC811 cathodes in contact with sulfide electrolytes can lead to the formation of resistive interphases that further exacerbate lithium-ion diffusion heterogeneity (*25*). Surface coatings emerge as a compelling solution to tackle these challenges. Coating layers act as a protective barrier to suppress undesirable side reactions with the LPSCl sulfide electrolyte and enhance the mechanical integrity of the cathode by reinforcing its structure (*39*–*42*). Recognizing this potential, we deposit a LNO coating on NMC811 cathodes. This approach leverages LNO’s dual functionality: enhancing the mechanical robustness of the cathode to resist stress-induced cracking and improving interfacial chemistry to suppress side reactions and reduce interfacial resistance.

**Fig. 4.**
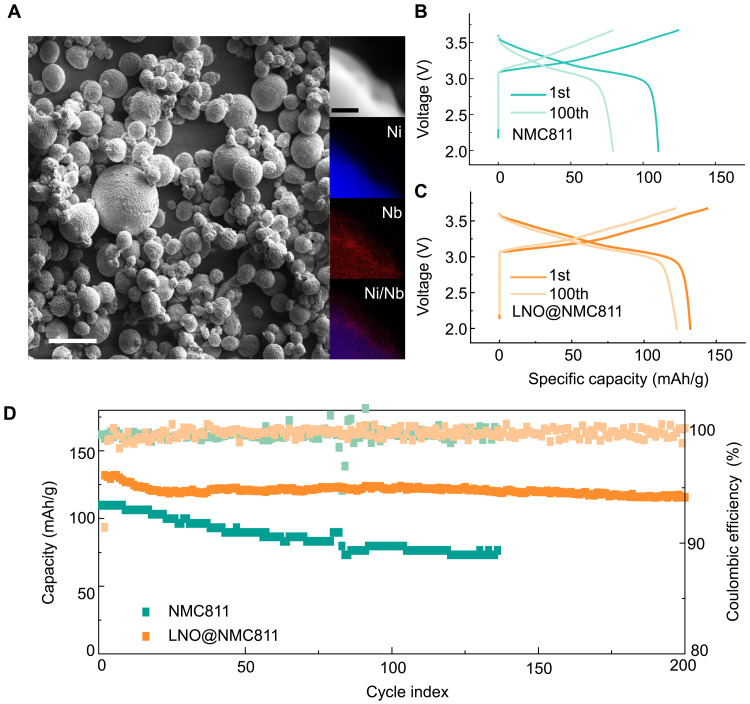
Physical properties and electrochemical performance evaluation of NMC811 and LNO@NMC811 cathodes. (**A**) SEM and high-angle annular dark-field images of LNO@NMC811, along with EDS elemental mapping. Scale bars, 50 and 200 nm. (**B**) Charge-discharge profiles of NMC811 cathodes at the first and 100th cycles. (**C**) Charge-discharge profiles of LNO@NMC811 cathodes at the first and 100th cycles. (**D**) Cycling performance and coulombic efficiency comparison of NMC811 and LNO@NMC811 cathodes over 200 cycles.

Scanning electron microscopy (SEM) images reveal that the NMC811 particles maintain their micron-sized secondary particle structure, indicating that the coating process does not induce substantial changes in particle shape or size (Fig. 4A). Scanning transmission high-angle annular dark-field imaging combined with energy dispersive x-ray spectroscopy (EDS) elemental distribution mapping indicates the presence of a clearly defined Nb-containing coating on the particle surface, confirming the successful surface modification (more information can be found in figs. S2 and S3). Figure 4 (B and C) displays the charge-discharge profiles of NMC811 and LNO@NMC811 cathodes. The LNO@NMC811 cathode shows a discharge capacity of 133 mAh/g in the first cycle, which decreases to 116 mAh/g after 200 cycles, demonstrating stable cycling performance. In contrast, the uncoated NMC811 cathode starts with a limited capacity of only 110 mAh/g and exhibits continuous degradation thereafter. Figure 4D highlights the capacity retention and coulombic efficiency throughout the entire cycling process, showing that the surface coating substantially reduces capacity fade and improves coulombic efficiency. Additional electrochemical performance data of the LNO@NMC811 samples can be found in figs. S4 to S6. The enhanced electrochemical performance that could attribute to LNO coating provides mechanical reinforcement against particle cracking and chemically stabilizes the interface by suppressing side reactions, promoting stable surface formation and alleviating space charge effects to enhance Li^+^ transport kinetics, as evidenced by the electrochemical impedance spectroscopy and distribution of relaxation times analysis in figs. S7 and S8.

**Fig. 5.**
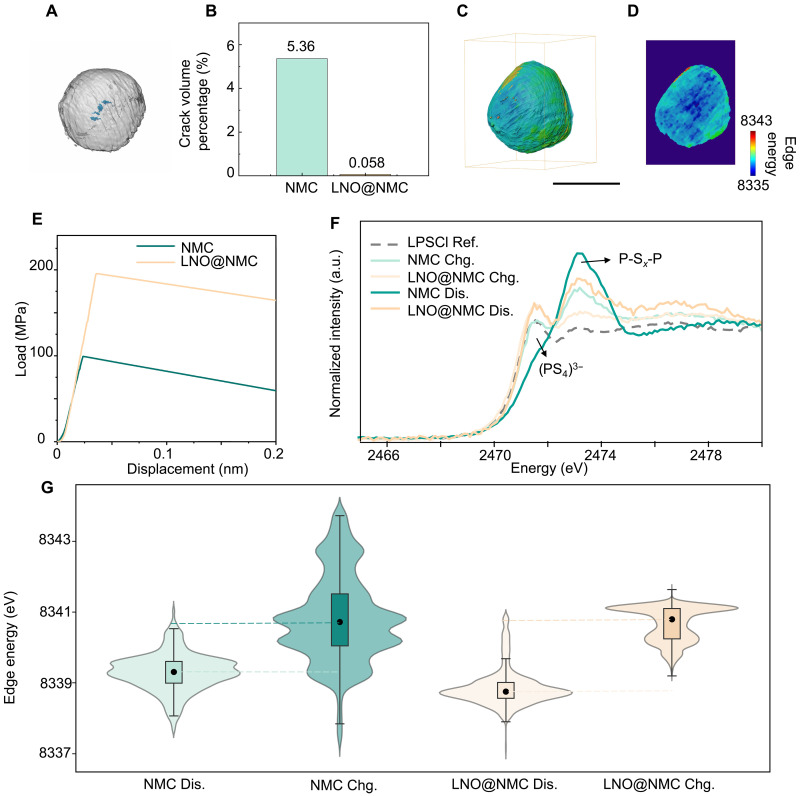
Mitigation of electro-chemo-mechanical interplay-induced capacity loss by LNO coating. (**A**) 3D volume rendering of cracks in LNO@NMC811 after cycling. (**B**) Comparison of crack volume ratio between LNO@NMC811 and bare NMC811 after cycling. (**C** and **D**) 3D rendering (C) and a slice (D) Ni K-edge absorption spectra imaging of the LNO@NMC811 particle [same as (A)]. (**E**) Compression tests of two10-μm NMC811 and LNO@NMC811 particles, highlighting the impact of the LNO coating on enhancing structural integrity. (**F**) S K-edge x-ray absorption spectroscopy for NMC811 and LNO@NMC811 at different states of charge after 100 cycles. (**G**) Violin plot of TXM-XANES results for charge (Chg.) and discharge (Dis.) states after the sixth cycle. (A), (C), and (D) share the scale bar. Scale bar, 10 μm. Ref, reference sample.

To further investigate and validate the mechanism of the LNO coating, postcycling LNO@NMC811 cathode particles were analyzed using TXM. Figure 5A shows the crack rendering of an LNO@NMC811 particle, obtained after one formation cycle at 0.05 C and five additional cycles at 0.1 C, revealing only a few small, noncontinuous cracks within the particle. In contrast, Fig. 5B highlights the substantial difference in crack severity: Postcycling, uncoated NMC811 particles exhibited a crack volume ratio of 5.36%, whereas LNO@NMC811 particles demonstrated a markedly reduced ratio of just 0.058%. Further evidence of crack evolution can be found in figs. S9 and S10. For the purpose of this analysis, features identified as cracks are those that develop during electrochemical cycling, as the pristine NMC811 particles are presumed to be initially dense. Figure 5 (C and D) presents Ni K-edge energy spectrum renderings, showing that LNO@NMC811 particles maintain a more uniform oxidation state distribution than uncoated NMC. Uniaxial compression tests of individual NMC811 and LNO@NMC811 particles further validates this effect. Figure 5E presents test results for two 10-μm-sized NMC811 and LNO@NMC811 particles, which shows higher fracture strength (estimated as the load at fracture divided by the cross-sectional area) for the coated NMC811 particle than the uncoated NMC811. This enhancement could help mitigate crack formation during cycling. On average, the coated NMC811 particles have a high fracture strength than the uncoated NMC811 particles (fig. S11), although there is a substantial distribution in values. This may be due to uneven LNO coatings on the NMC811 particles.

Tender x-ray absorption spectroscopy was used to investigate the sulfur chemistry at the cathode/electrolyte interface for both NMC811 and LNO@NMC811 samples, collected after 100 full cycles, across different SOC. The S K-edge x-ray absorption spectroscopy (Fig. 5F) revealed notable changes in the interphase sulfur electronic state. For cycled NMC811 samples, the intensity of the peak at 2472.0 eV, attributed to bridging sulfur (P─S*_x_*─P) bonds (*43*), increased, suggesting notable interfacial reactions between LPSCl solid electrolytes and NMC811 cathodes. Particularly in the charged state, the PS_4_^3−^ tetrahedral fingerprint spectrum disappeared, indicating oxidation and severe degradation of the LPSCl solid electrolyte. In contrast, LNO@NMC811 cathodes showed a more stable interface, with fewer side reactions and suppressed chemical degradation. The improved chemical uniformity is further illustrated in the 2D absorption spectra images (fig. S12). Uncoated NMC811 particles displayed a pronounced increase in oxidation state after 100 cycle cycling, particularly in the charged state, with a broad distribution of oxidation states (Fig. 5G). In contrast, LNO@NMC811 particles exhibited a narrower and more uniform oxidation state distribution. Moreover, the median difference in Ni oxidation states between charge and discharge for LNO@NMC811 was greater, indicating a higher degree of electrochemical activity. In addition, micron-scale x-ray fluorescence (XRF) mapping and spectroscopy were conducted at SSRL beamline 2-3 to validate this, with the experimental setup shown in fig. S13. The results (fig. S14) indicate that the chemical heterogeneity of the LNO@NMC811 sample is reduced. Compared to the NMC811 sample, the Ni oxidation state in the charged state is higher and more homogeneous providing more capacity for electrochemical reactions. Synchrotron x-ray diffraction (XRD) experiments were conducted at beamline 11-3 of SSRL in transmission mode, where the x-ray beam penetrated through the sample pellets consisting of NMC811 cathode, electrolyte, anode, and current collector. The diffraction patterns are shown in fig. S15. A zoom-in of the (003) reflections (fig. S16) reveal a progressive shift toward lower angles with increasing cycle numbers for the NMC811 samples, which can be attributed to increasingly incomplete lithium reinsertion in the discharged state, as evident from the refined lattice parameters (summarized in fig. S17). Concurrently, notable peak broadening is observed, likely due to the accumulation of lattice strain and intergranular heterogeneity. These structural degradations are notably suppressed in the LNO-coated samples, indicating the coating’s effectiveness in mitigating mechanical and structural degradation. This enhanced uniformity and reduced heterogeneity in lithium transport highlight the LNO coating’s ability to suppress electro-chemo-mechanical degradation, leading to better stability and improved electrochemical performance.

## DISCUSSION

Using advanced x-ray imaging techniques, we investigate the failure mechanisms of NMC811 cathodes in sulfide-based SSBs. Our findings reveal the critical role of electro-chemo-mechanical interactions in cell degradation, which are far more substantial than previously anticipated. Uneven lithium-ion transport induces spatially heterogeneous electrochemical reactions, mechanical stress, and intergranular cracking, creating isolated domains that hinder lithium-ion diffusion and cause capacity loss. These factors further exacerbate SOC heterogeneity, triggering a vicious cycle of harmful mechanical degradation. To address these challenges, we revisit an LNO coating strategy for NMC811. The LNO coating serves as a chemically stable barrier that suppresses parasitic interfacial reactions and facilitates efficient Li^+^ transport across the cathode electrolyte interface. In addition, its mechanical robustness mitigates stress-induced cracking during cycling, thereby preserving the structural integrity of the NMC811 cathode (fig. S18). The LNO@NMC811 cathode delivers an initial discharge capacity of 133 mAh/g, which only slightly decreases to 116 mAh/g after 200 cycles, highlighting its excellent cycling stability. In sharp contrast, the uncoated NMC811 cathode exhibits a lower initial capacity of merely 110 mAh/g and suffers from continuous capacity fading throughout cycling. The state-of-the-art imaging and spectroscopic analyses confirm that the coating mitigates interfacial reactions and enhances the structural integrity of the cathode, effectively suppressing crack formation. Our work underscores the pivotal role of advanced imaging and characterization techniques in uncovering the complex electrochemical-mechanical interplay within SSBs. Continued advancement in these methodologies could refine our understanding of failure mechanisms and support the development of more robust materials and interface designs.

## MATERIALS AND METHODS

### Materials and chemicals

LPSCl film was fabricated using a slurry casting method reported previously. Poly(isobutylene) binder (with a molecular weight of 1270 kg mol^−1^) and argyrodite were milled in a weight ratio of 5:95. Toluene, accounting for 46 wt % of the total slurry weight was used as the solvent. The slurry was ball-milled during a low-velocity mixing step with zirconia milling media overnight (≥18 hours). After milling, the slurry was cast onto a sheet of nonstick Mylar and allowed to dry at room temperature before being sealed in a pouch between two Mylar sheets for calendaring outside the glove box. Calendaring was performed using a cold roller press (MTI cold roller press, MSK-HRP-MR100DC) at room temperature. A composite cathode was prepared by dry milling a mixture in a ratio of 60:35:5 of NMC811 active materials (with or without LiNbO_3_ coating, both acquired from NEI Corp.), LPSCl, and vapor growth carbon fiber in a turbula mixer for 1 hour.

### Electrochemical measurements

Six-millimeter-diameter polyether ether ketone (PEEK) mode cells (Solid Solutions, 7815-C6) were deployed for testing cycling performance at ambient temperature within an argon-filled glove box. Two discs of LPSCl film were slowly placed in PEEK mode, and 20 bar was applied for 10 s to densify the film. Five-milligram power of composite cathode and carbon-coated aluminum foil was added on top of LPSCl film, and 500 MPa fabrication pressure was applied for 3 min. A 5-mm-diameter indium foil (150 × 150 TF, Custom Thermoelectric) and a 6-mm-diameter copper foil were positioned in the cell as the anode. Galvanostatic cycling was performed with three-formation cycle at 0.05 C and extensively cycle at 0.1 C under a stack pressure of 10 MPa. Electrochemical impedance spectroscopy (EIS) was measured from 1 MHz to 50 mHz with 10-mV amplitude, and corresponding distribution of relaxation times (DRT) analysis was derived using the pyDRTtools package.

### Characterization

The TXM experiments were conducted at beamline 6-2c of SSRL. NMC811 particles were carefully shaved from the electrode and loaded into a capillary inside an Ar-filled glove box. Both ends of the capillary were sealed with epoxy to ensure airtightness. The capillary was mounted on the sample holder and positioned perpendicular to the incident x-ray beam. Nanotomography data were collected by rotating the sample holder from −90° to 90° with a 1° step size. Ni K-edge tomography was acquired by capturing projection images during an energy scan from 8100 to 8800 eV in approximately 80 steps. The energy range was sampled at steps of 10 or 20 eV before and after the absorption edge and at 1-eV steps around the absorption edge. Each image was taken with a 2-s exposure time (binning, 2; resolution, 1024 × 1024 pixels). Data analysis was carried out using the in-house software TXM-Wizard (*34*). The XRF microprobe and x-ray absorption spectroscopy experiments were conducted at Beamline 2-3 and 14-3b of the SSRL (SLAC National Accelerator Laboratory). An axially symmetric Sigray focusing optics was used to focus the x-ray beam to a spot size of ~1 μm. First, XRF was used to locate the particles of interest, after which the x-ray beam was focused on several characteristic points on the particle, either at the edges or the center. Energy scans were performed in the 8100- to 8600-eV range for beamline 2-3 and in the 2400- to 2500-eV range for Beamline 14-3b, with fluorescence data collected by positioning a Vortex detector at a 45° angle to the sample. The fluorescence data were analyzed using the in-house developed SMAK package (*44*). The particle compression tests were performed using an iMicro nanoindenter (KLA Instruments) equipped with a diamond flat punch of 55 μm in diameter (Synton-MDP). First, the particles were dispersed onto silicon wafers via the dry process. Then, each particle was compressed and unloaded at constant displacement rates of 10 and 100 nm s^−1^, respectively, and the data acquisition rate was 400 Hz. Synchrotron XRD with 12.7-keV x-ray energy in transmission mode was conducted on the harvested sample pellets, consisting of the cathode and LPSCl solid electrolyte for the pristine samples and additionally the In anode and Cu current collector for the cycled samples. Data integration was done using pyFAI (*45*). Sample-to-detector distance was determined by combining information from LaB_6_ as an external reference and LPSCl as an internal reference. Pawley refinement on the XRD data was performed using Topas-Academic V7 (*46*). Instrument broadening was estimated on the basis of LaB_6_ data. Microstrain broadening of the samples were treated by Pseudo-Voigt convolutions. SEM images of cross sections were taken following focused ion beam (FIB) milling on an FEI Helios NanoLab 600i DualBeam scanning electron microscope, an FIB system (Ga+), and plasma focused ion beam scanning electron microscope (Xe+). The initial coarse trenching was executed at an accelerating voltage of 30 kV and beam currents of 20 to 60 nA. This was followed by a stepwise polishing, with beam currents progressively reduced from 9 nA down to 0.79 nA to minimize amorphization and “curtaining.”
