# Supplementary material for: Unravelling electro-chemo-mechanical interplay in layered oxide cathode degradation in solid-state batteries
Source: Sci Adv. 2025 Oct 8;11(41):eady7189. doi: 10.1126/sciadv.ady7189 (PMC12506959; doi:10.1126/sciadv.ady7189)
Supplement: Supplementary file 1 — Figs. S1 to S18 [file sciadv.ady7189_sm.pdf]

Science Advances
AAAS

Supplementary Materials for

Unravelling electro-chemo-mechanical interplay in layered oxide cathode degradation in solid-state batteries

Xueli Zheng *et al.*

Corresponding author: Jagjit Nanda, jnanda@slac.stanford.edu; Guang Yang, yangg@ornl.gov; Xueli Zheng, xuelizh8@stanford.edu; Zhichen Xue, slacxue@stanford.edu

Sci. Adv. **11**, eady7189 (2025)
DOI: 10.1126/sciadv.ady7189

This PDF file includes:

Figs. S1 to S18

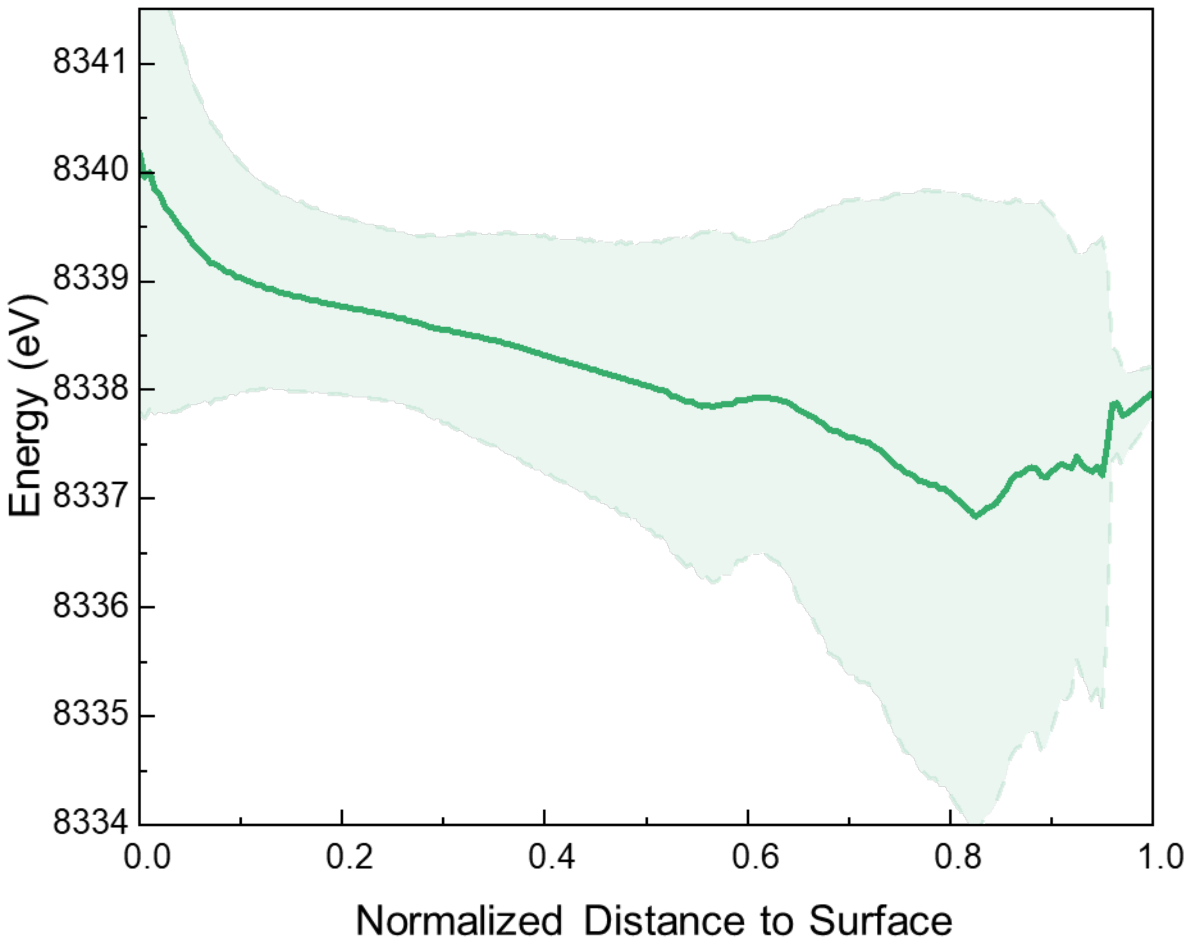

Fig. S1 Depth-dependent variation in edge energy reveals surface-dominated side reactions and bulk heterogeneity.

Depth profile of the averaged edge energy shift (green line) and the corresponding standard deviation (shaded area). Significant edge shifts and increased variation are observed near the surface region, indicating strong surface side reactions at high SOC. In contrast, within the bulk (lower SOC), the gradual increase in edge position and variation implies valence state elevation and increased heterogeneity, likely due to isolated electrochemically active domains.

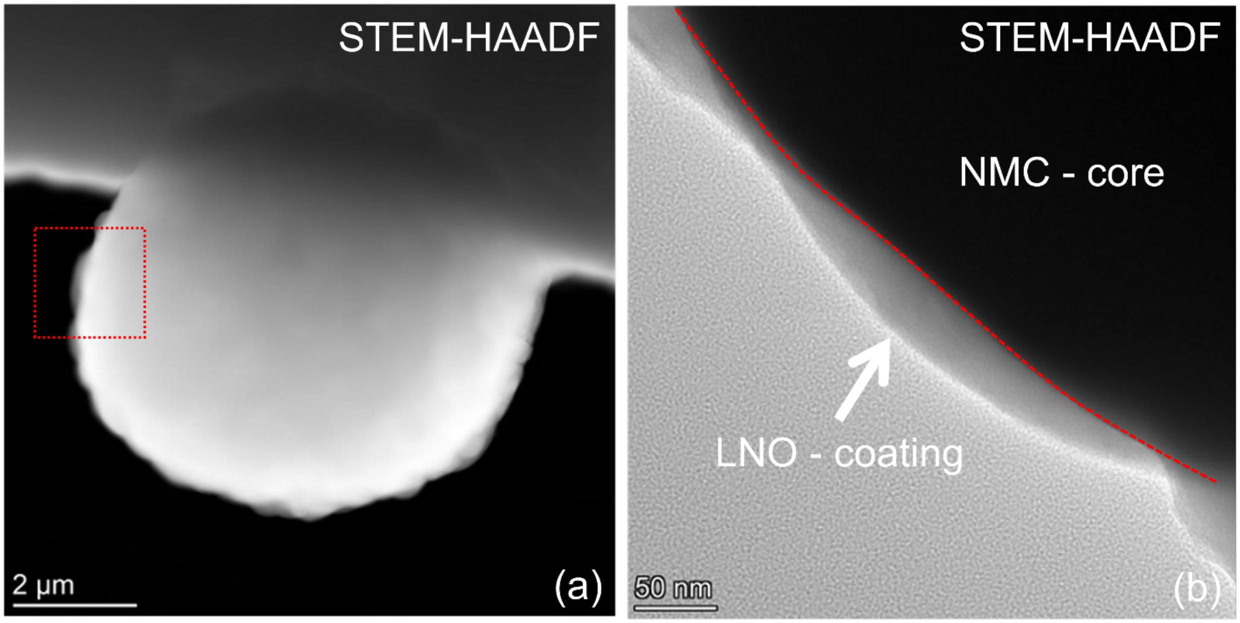

Fig. S2 STEM image reveals the coherent interface between the LNO coating and NMC core.

Scanning transmission electron microscopy (STEM-HAADF) image of the LNO@NMC cathode, showcasing the interface between the LNO coating layer and the NMC core.

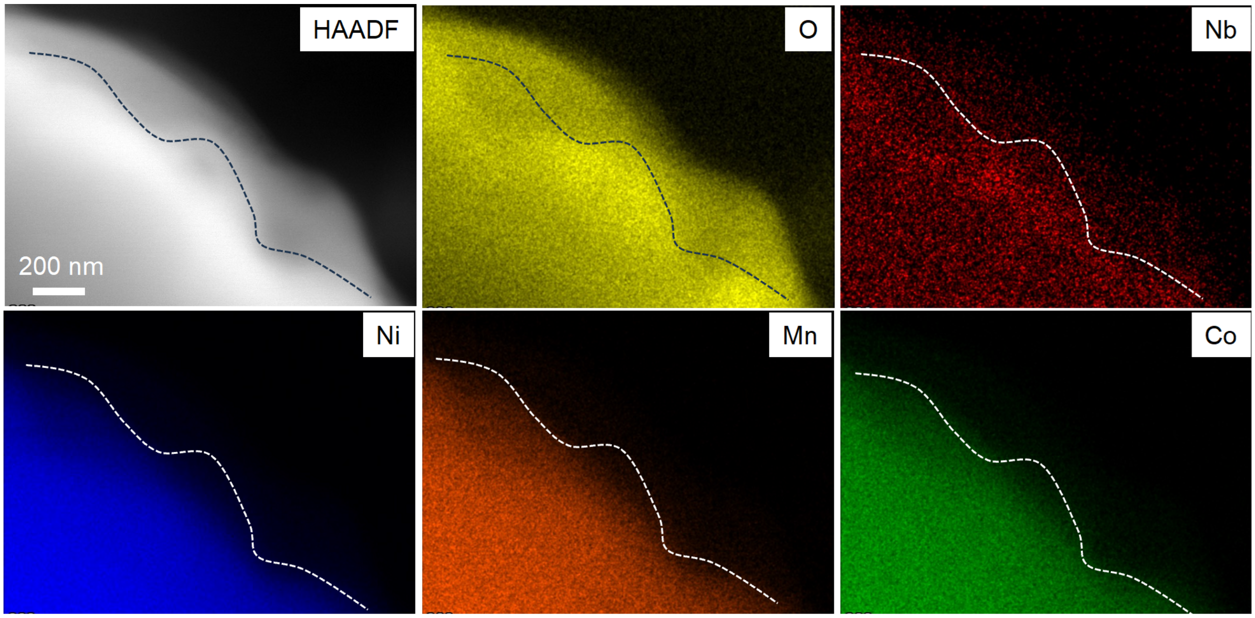

Fig. S3 Elemental mapping confirms conformal LNO coating on the NMC cathode surface. STEM-HAADF image (top left) of the LNO@NMC cathode and the corresponding energy dispersive spectroscopy (EDS) elemental distribution maps, showcasing the interface between the LNO coating layer and the NMC core.

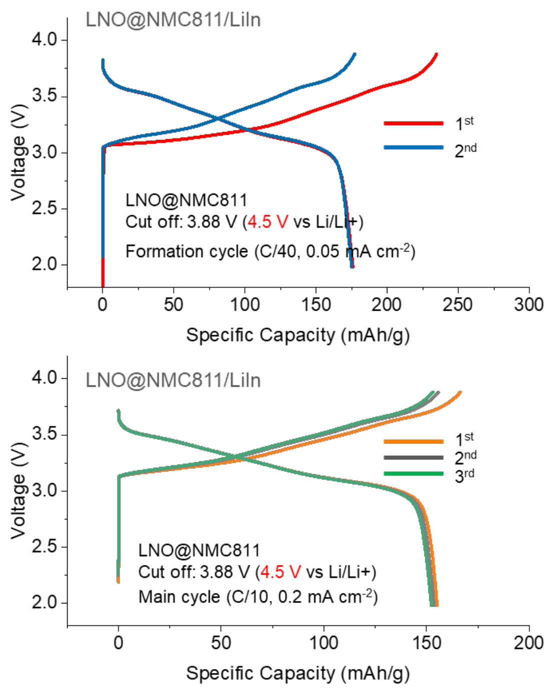

Fig. S4 High Voltage cutoff influences cycling behavior of LNO@NMC811‖LiIn cells. Voltage profiles of LNO@NMC811‖LiIn cells during the first five cycles under different voltage cutoffs. The top panels show the initial two formation cycles at C/40, and the bottom panels show the subsequent three cycles at 0.1C. Left: cells cycled to 4.5 V; right: cells cycled to 4.7 V.

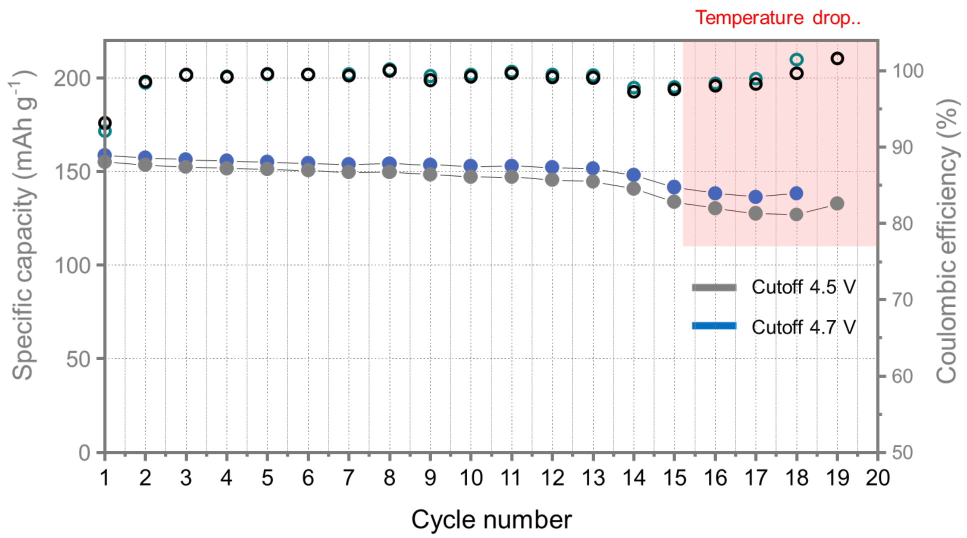

Fig. S5 Long-term cycling stability of LNO@NMC811 under high-voltage conditions. Cycling performance of LNO@NMC811 cells. Cells were cycled at 0.1C under high-voltage conditions (charged to 4.5 V and 4.7V). The LNO@NMC811 demonstrates improved capacity retention and more stable Coulombic efficiency. The fluctuation in Coulombic efficiency observed in the later cycles (highlighted in red) is primarily attributed to a drop in ambient temperature, which affects the ionic conductivity of the solid electrolyte.

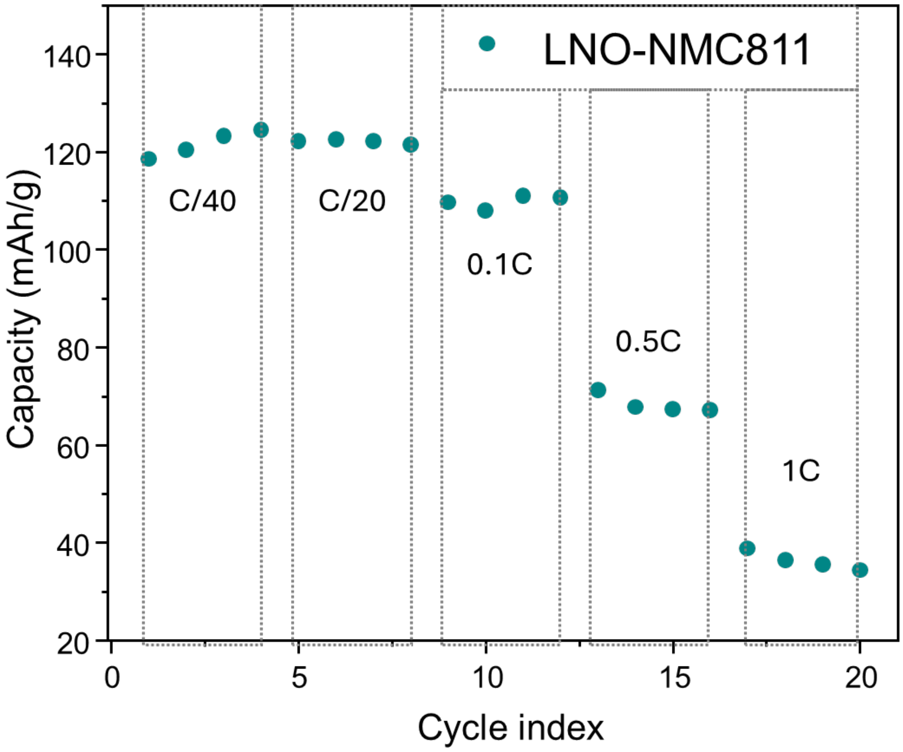

Fig. S6 Rate performance of LNO@NMC811 cells.

The specific capacity remains stable at low rates (C/40 and C/20). However, a noticeable capacity drop is observed at higher rates (⩾0.5 C), suggesting transport limitations likely associated with interfacial resistance and solid-state ion conduction constraints.

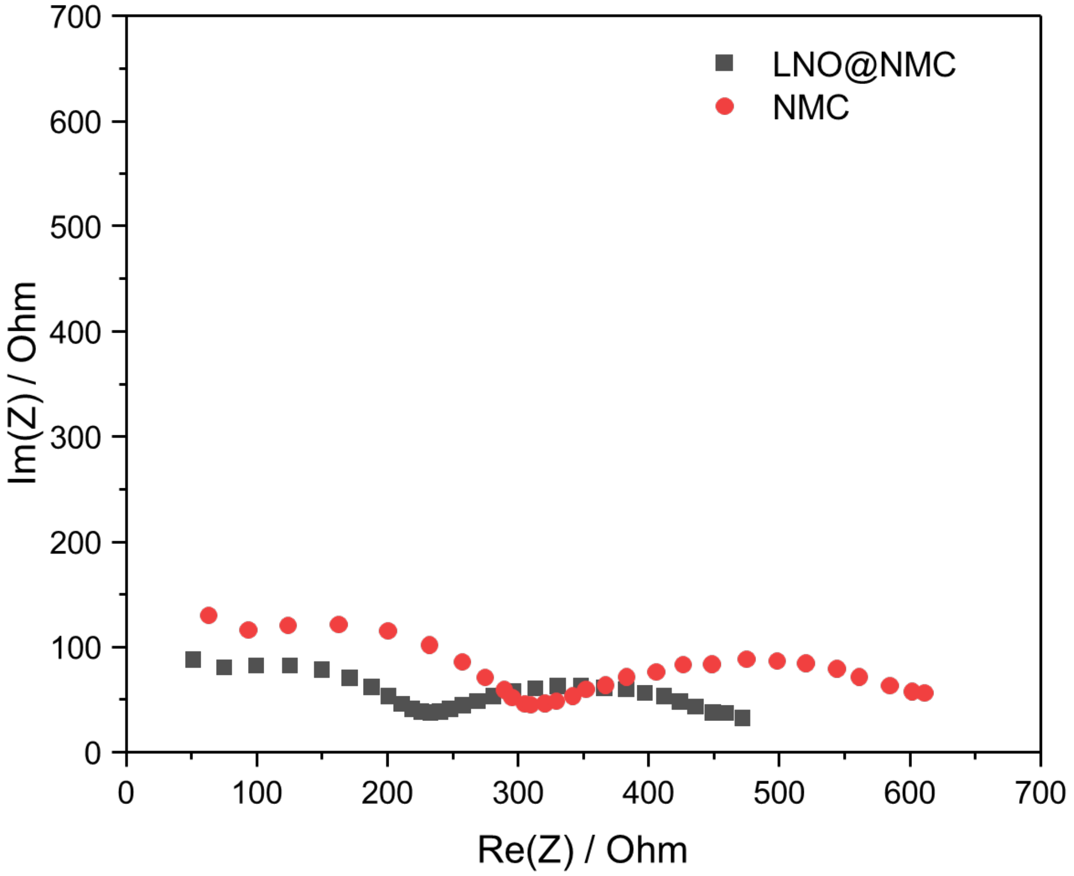

Fig. S7 Electrochemical impedance spectroscopy of NMC811 cells with and without LNO coating.

EIS was collected at the end of the 4th cycle after formation for both cells. The LNO@NMC811 exhibits a smaller semicircle in the high-to-mid frequency range, indicating reduced interfacial resistance and improved Li^+ transport compared to the NMC sample.

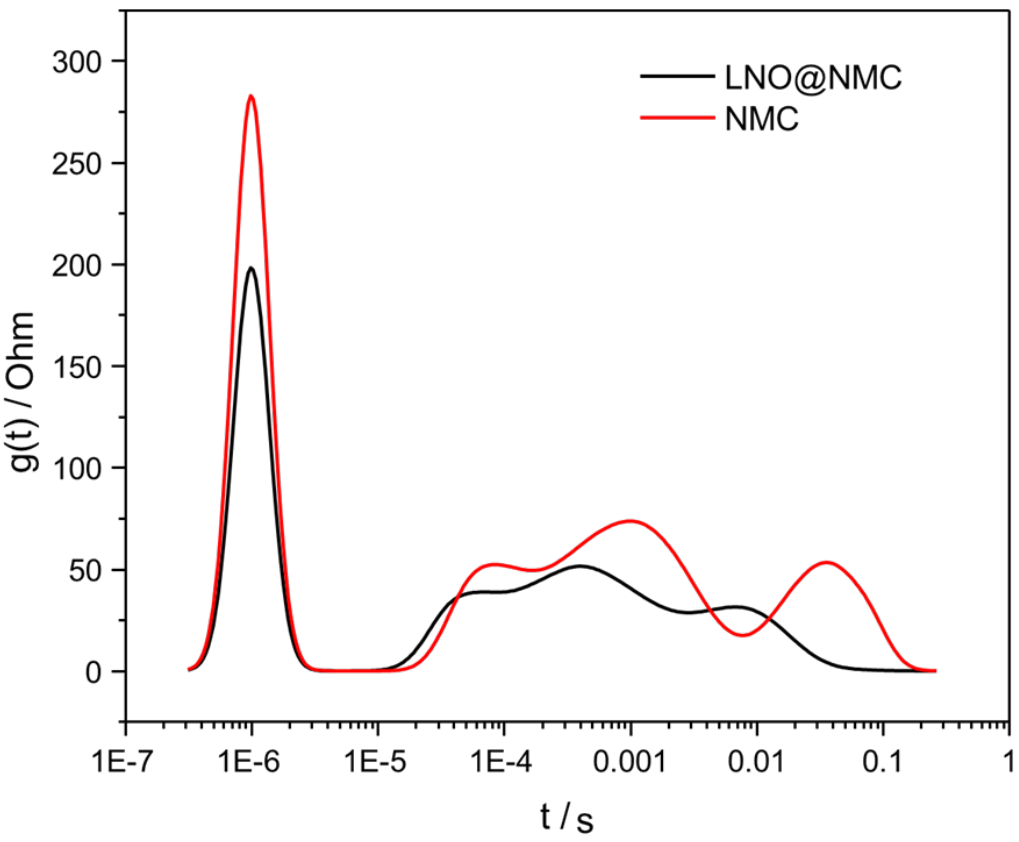

Fig. S8 Distribution of relaxation times (DRT) analysis for NMC811 cells with and without LNO coating, based on EIS measurements collected at the end of the 4th cycle.
The LNO-coated NMC811 (black) shows markedly lower impedance across multiple time domains compared to the uncoated sample (red), particularly in the high-frequency region ($\sim 10^{-6}$ s), which corresponds to bulk resistance. Finally, the charge-transfer resistance observed at longer timescales (10^{-2} to 10^{-1} s) likely reflects electronic transport limitations, potentially arising from interfacial degradation or the absence of a protective coating.

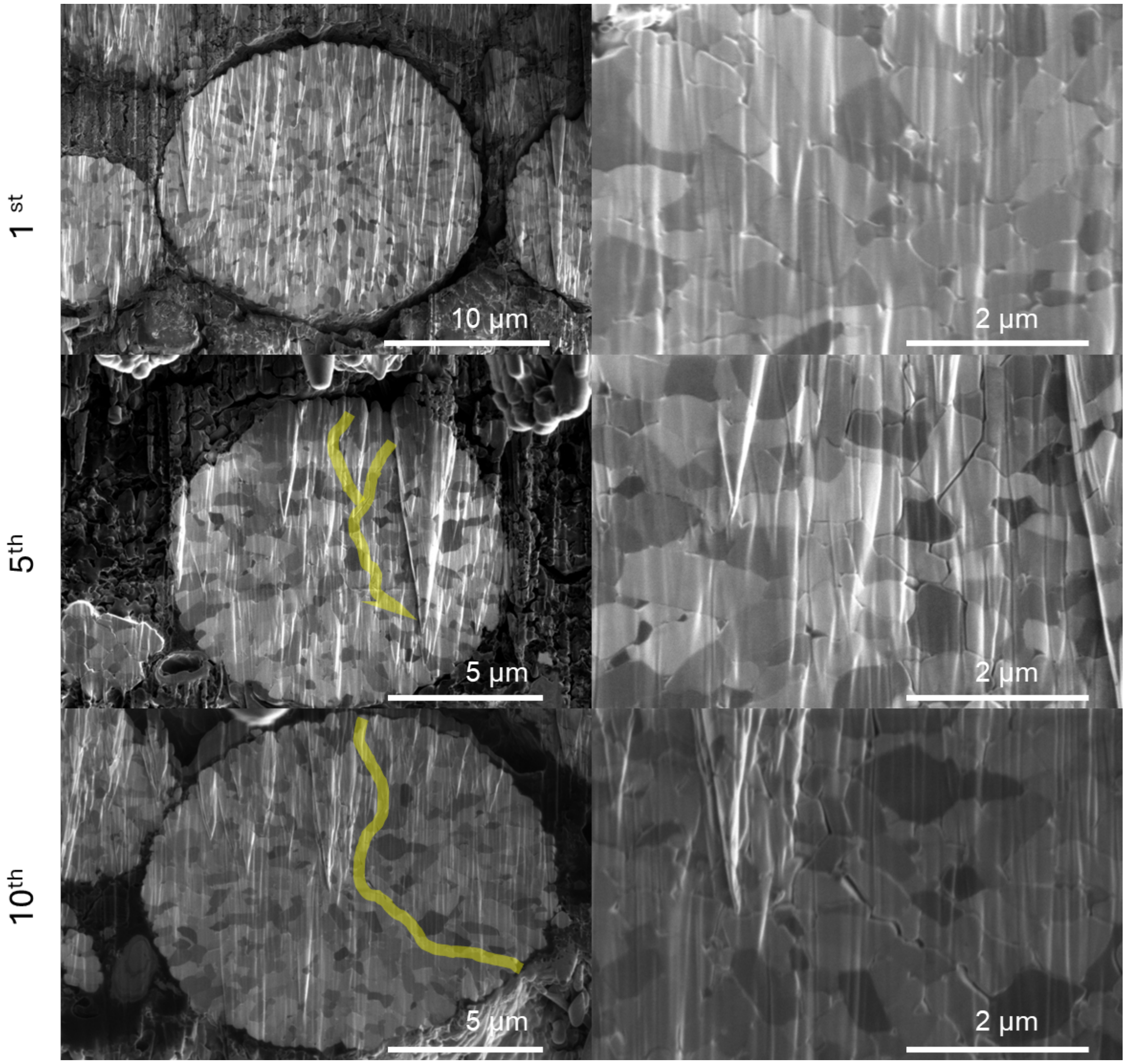

Fig. S9 Focused ion beam scanning electron microscopy (FIB-SEM) cross-sectional images of NMC811 particles after cycling.

Images correspond to the 1st, 5th, and 10th cycles (from top to bottom). No visible cracks are observed after the 1st cycle, whereas prominent intergranular cracks emerge by the 5th cycle. By the 10th cycle, the cracks have propagated throughout the entire particle, indicating progressive mechanical degradation during cycling.

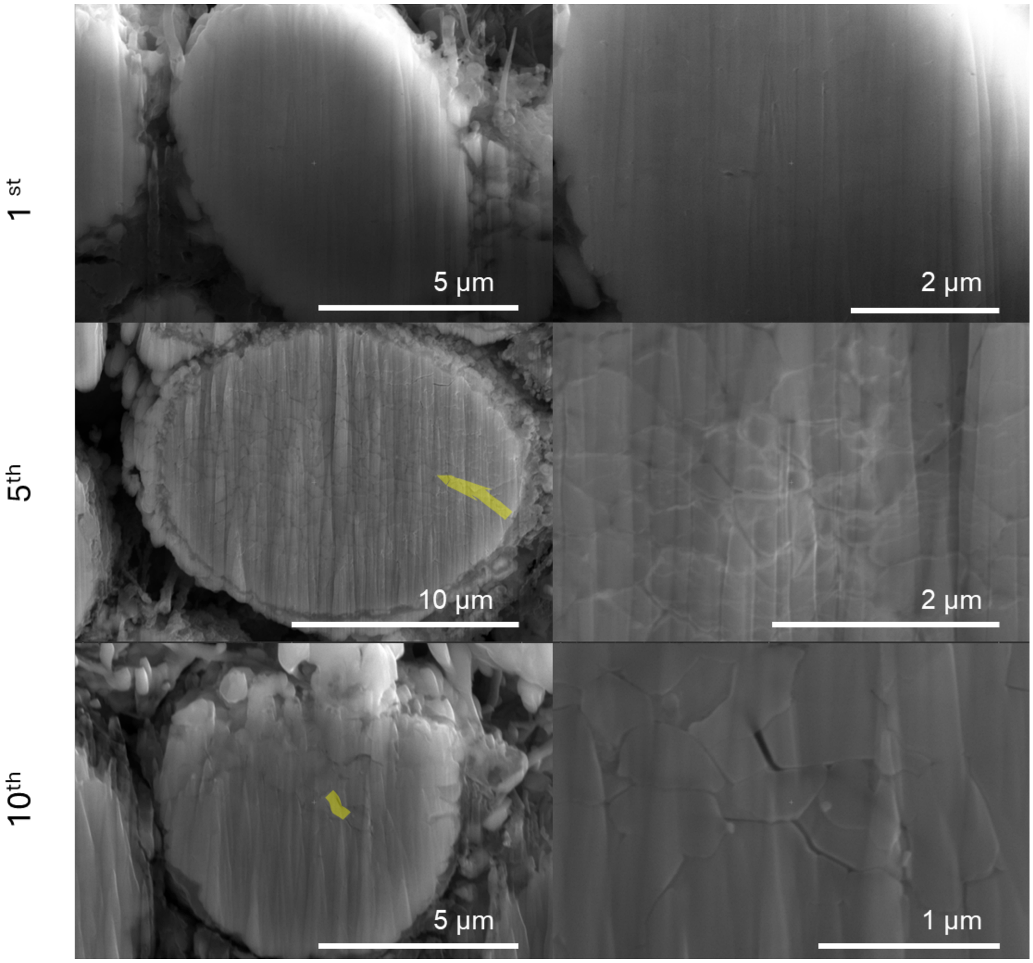

Fig. S10 FIB-SEM cross-sectional images of LNO@NMC811 particles after electrochemical cycling.

Images correspond to the 1st, 5th, and 10th cycles (from top to bottom). Compare to NMC811 sample, the LNO@NMC811 sample exhibits a crack-less structure, indicating that the coating effectively suppresses electrochemically induced mechanical degradation.

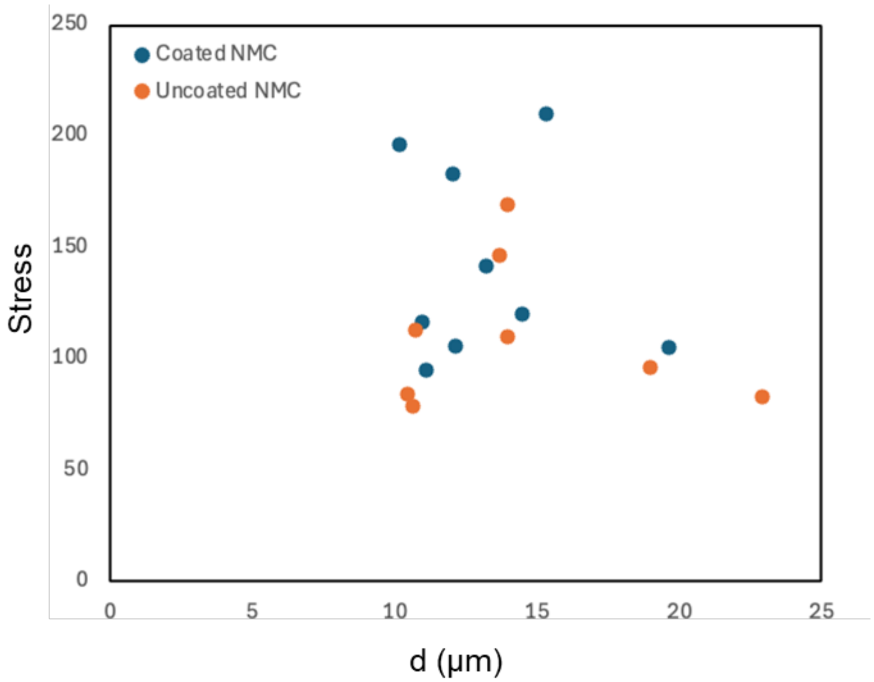

Fig. S11 Fracture strengths of LNO@NMC particles and uncoated NMC particles as a function of particle diameter (d).

Fracture strength is estimated as the fracture load divided by the cross-sectional area of the particle.

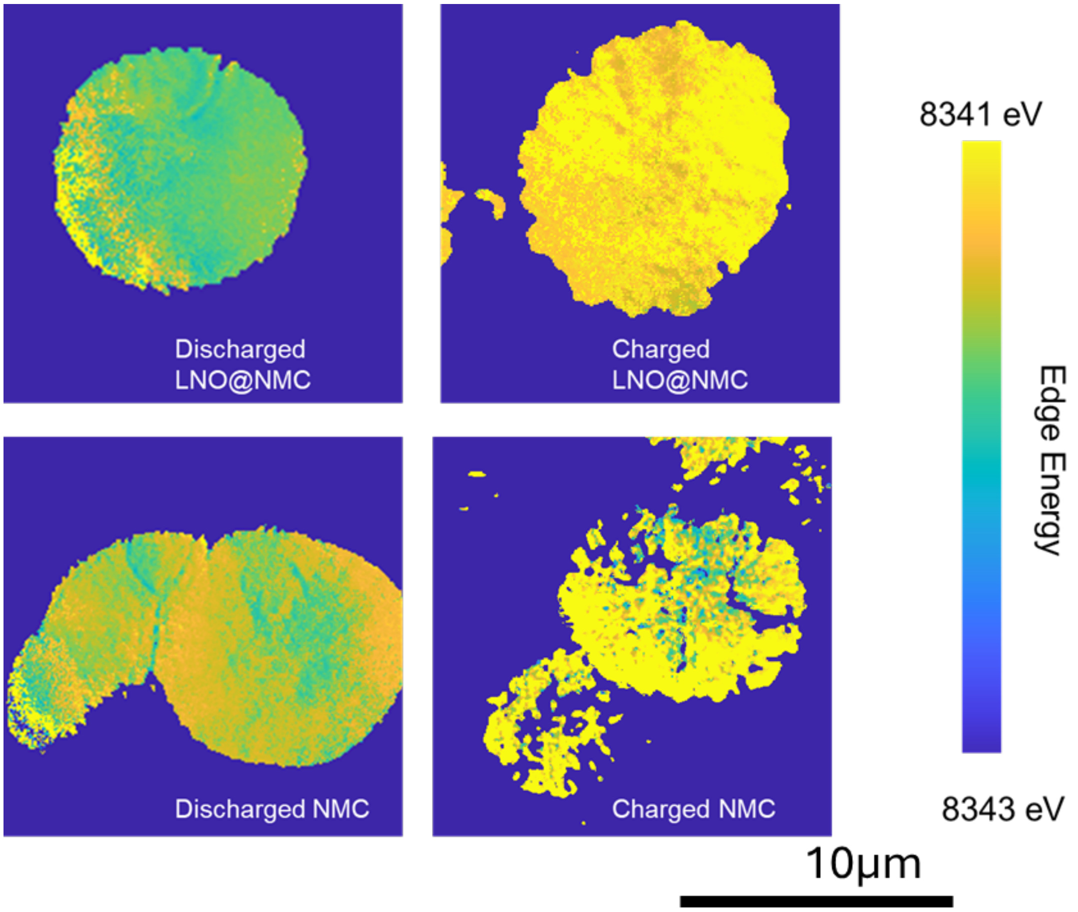

Fig. S12 2D TXM imaging combined with Ni K-edge X-ray absorption spectroscopy for NMC811 and LNO@NMC811 particles in their charged and discharged states.

The particles were cycled with one formation cycle at C/20 followed by five cycles at C/10. Charged and discharged particles are different, and all images are 2D projections. Notably, the discharged LNO@NMC811 particle is the same one shown in Figure 2, but here presented in projection, where internal cracks may appear more severe due to overlap along the projection axis.

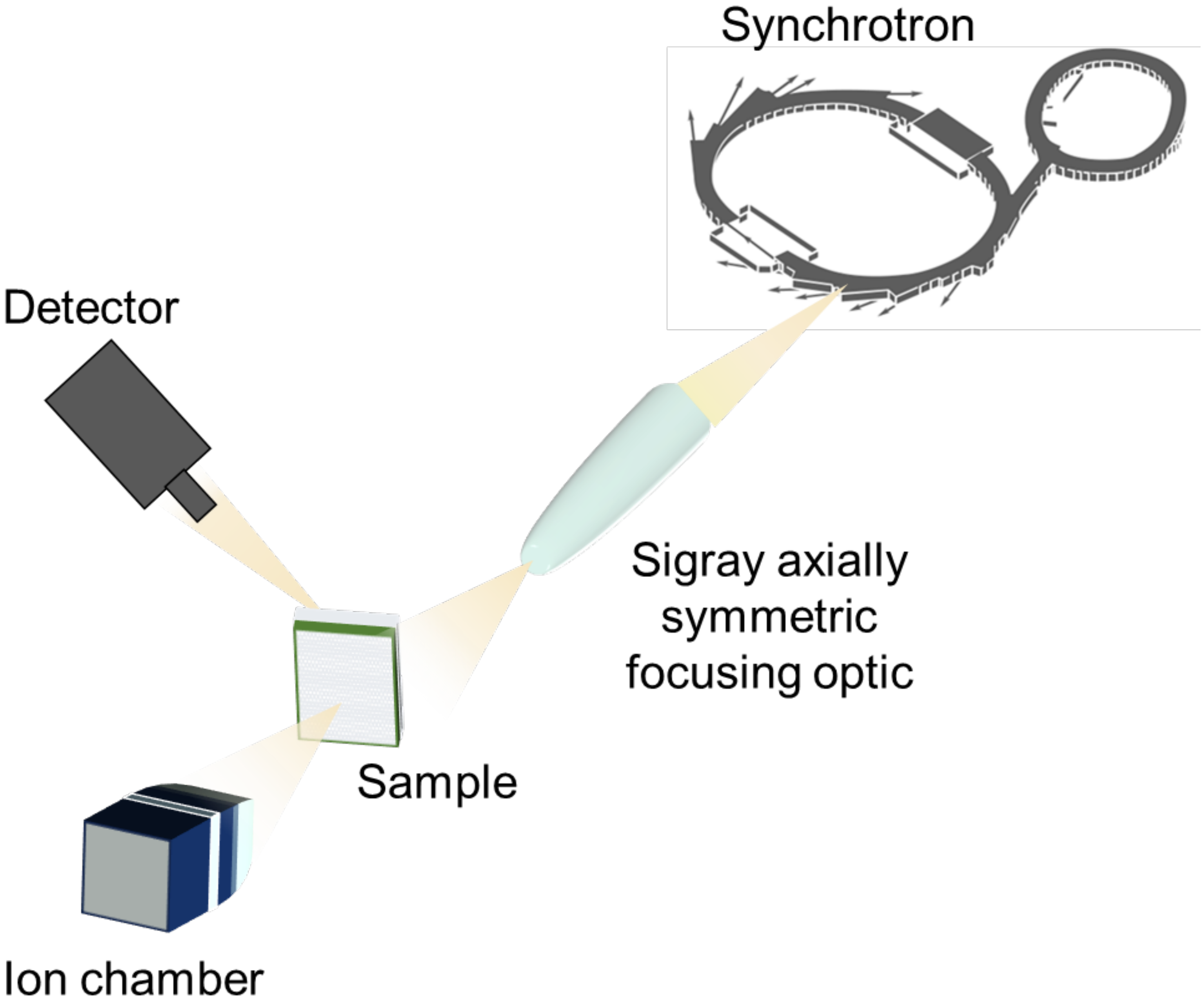

Fig. S13 Schematic of the experimental setup for micron-scale X-ray fluorescence and X-ray absorption near-edge structure spectroscopy.

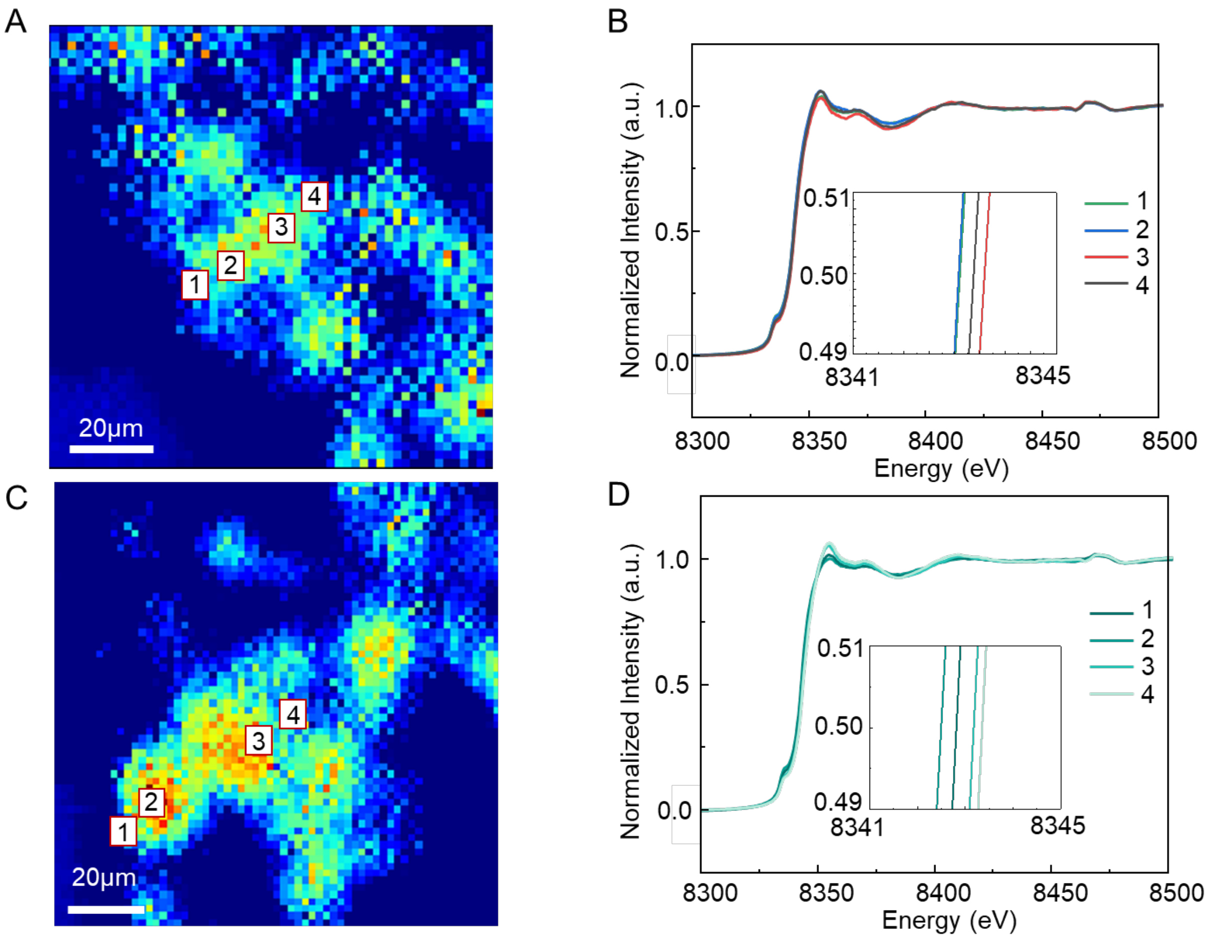

Fig. S14 Spatially resolved X-ray analyses reveal compositional and charge heterogeneity after cycling.

X-ray fluorescence mapping of the charge state of LNO@NMC811 (A) and NMC811 (C) after cycling, showing the spatial distribution of elements. B.D. The corresponding Ni X-ray absorption near-edge structure spectra for each point in the map.

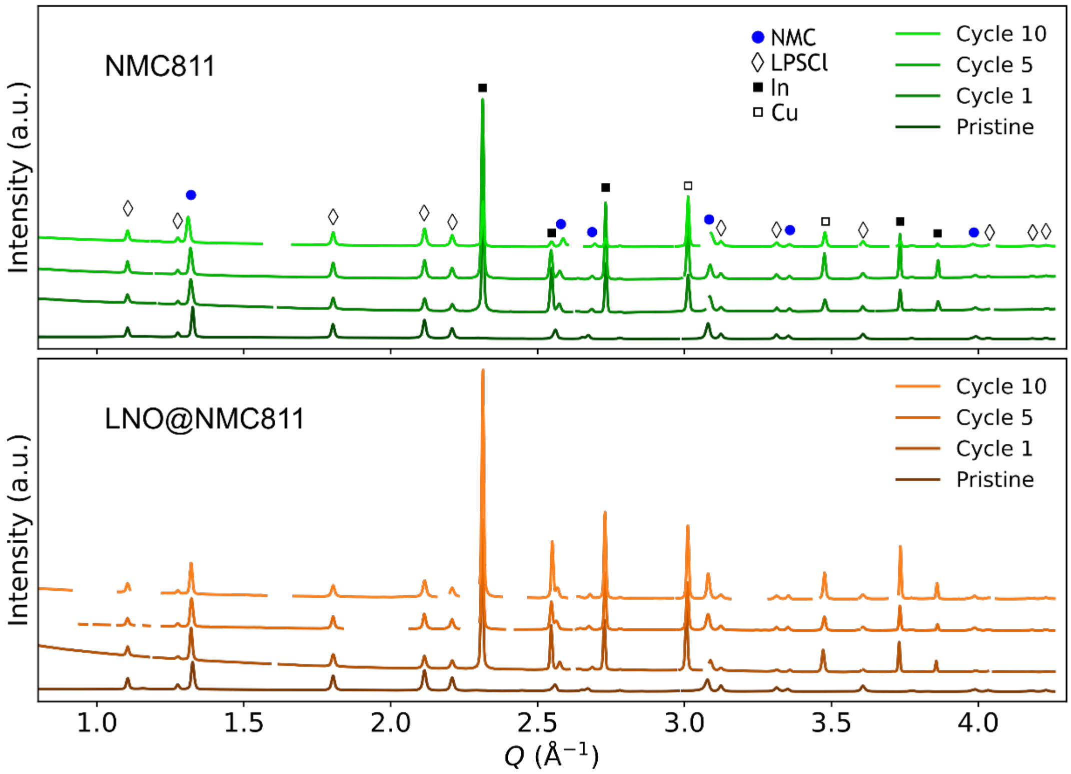

Fig. S15 Synchrotron XRD reveals structural evolution of NMC811 and LNO@NMC811 during cycling.

Synchrotron XRD patterns of NMC811 (top) and LNO@NMC811 (bottom) cells collected at pristine state, after 1, 5, and 10 cycles. X-ray wavelength: 0.9762535 Å. Phase identification for the peaks is shown. Artifact signals appeared inconsistently due to some bright spots recorded on the detector panel (unknown origin). The regions affected by these artifacts were excluded from the plot.

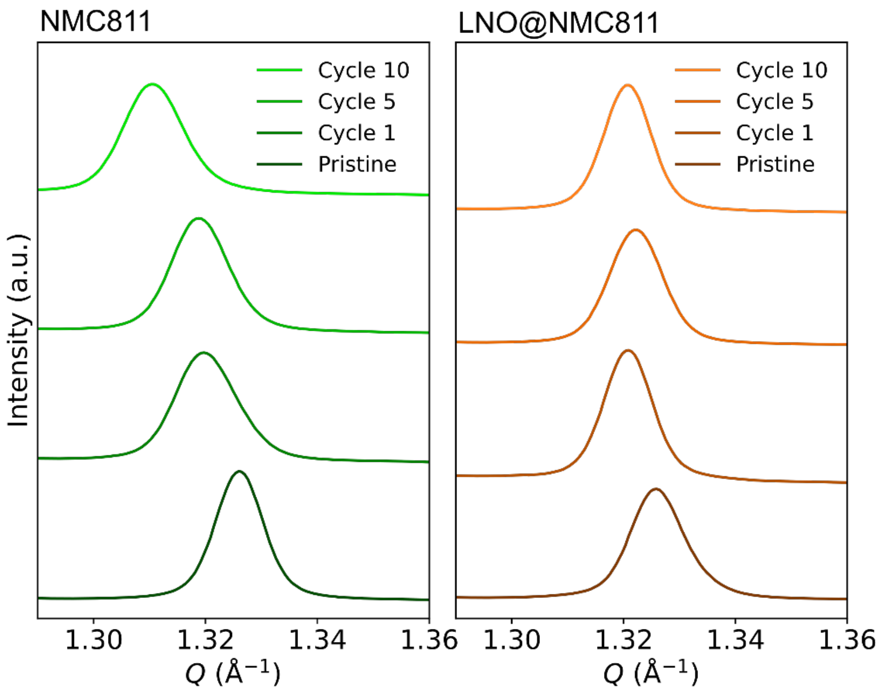

Fig. S16 LNO coating stabilizes the lattice structure of NMC811 during extended cycling.

Zoom-in view of the (003) reflection from ex situ synchrotron XRD patterns of NMC811 (left) and LNO@NMC811 (right) collected after different cycle numbers. With increasing cycle number, the NMC811 sample exhibits a clear shift towards lower angle and peak broadening, indicative of lattice parameter changes due to lithiation differences and the accumulation of strains. In contrast, the LNO-coated NMC811 shows more stable peak positions and narrower profiles, suggesting mitigated structural degradation and improved lattice integrity upon cycling.

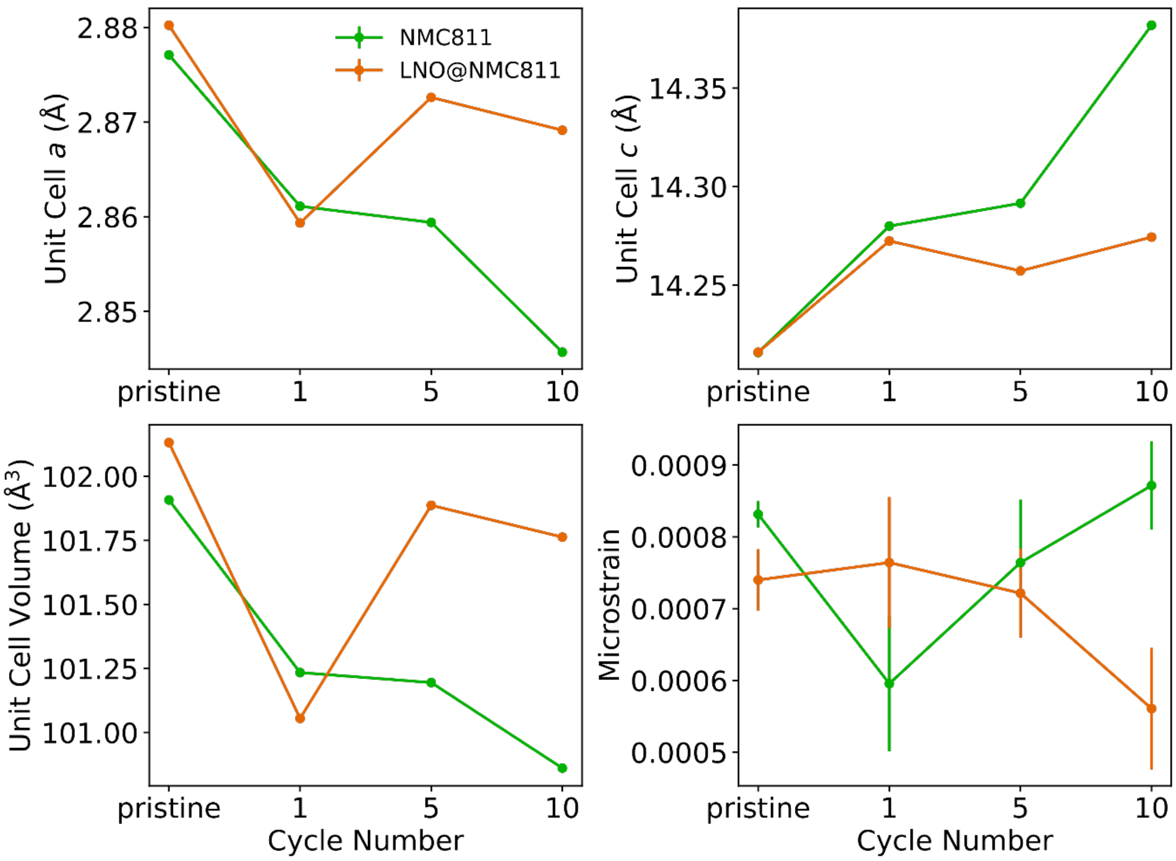

Fig. S17 LNO coating mitigates anisotropic lattice distortion and strain accumulation during cycling.

Evolution of structural parameters extracted from ex situ synchrotron XRD patterns of NMC811 and LNO@NMC811 electrodes at different cycle numbers (pristine, 1, 5, and 10). Top left: in-plane lattice parameter a; top right: out-of-plane lattice parameter c; bottom left: unit cell volume; bottom right: microstrain estimated from peak broadening. Upon cycling, the uncoated NMC811 exhibits a continuous decrease in a, increase in c, and growth in microstrain, suggesting anisotropic lattice distortion and strain accumulation. In contrast, the LNO-coated samples maintain more stable lattice parameters and suppressed microstrain evolution, highlighting the effectiveness of the coating in mitigating structural degradation.

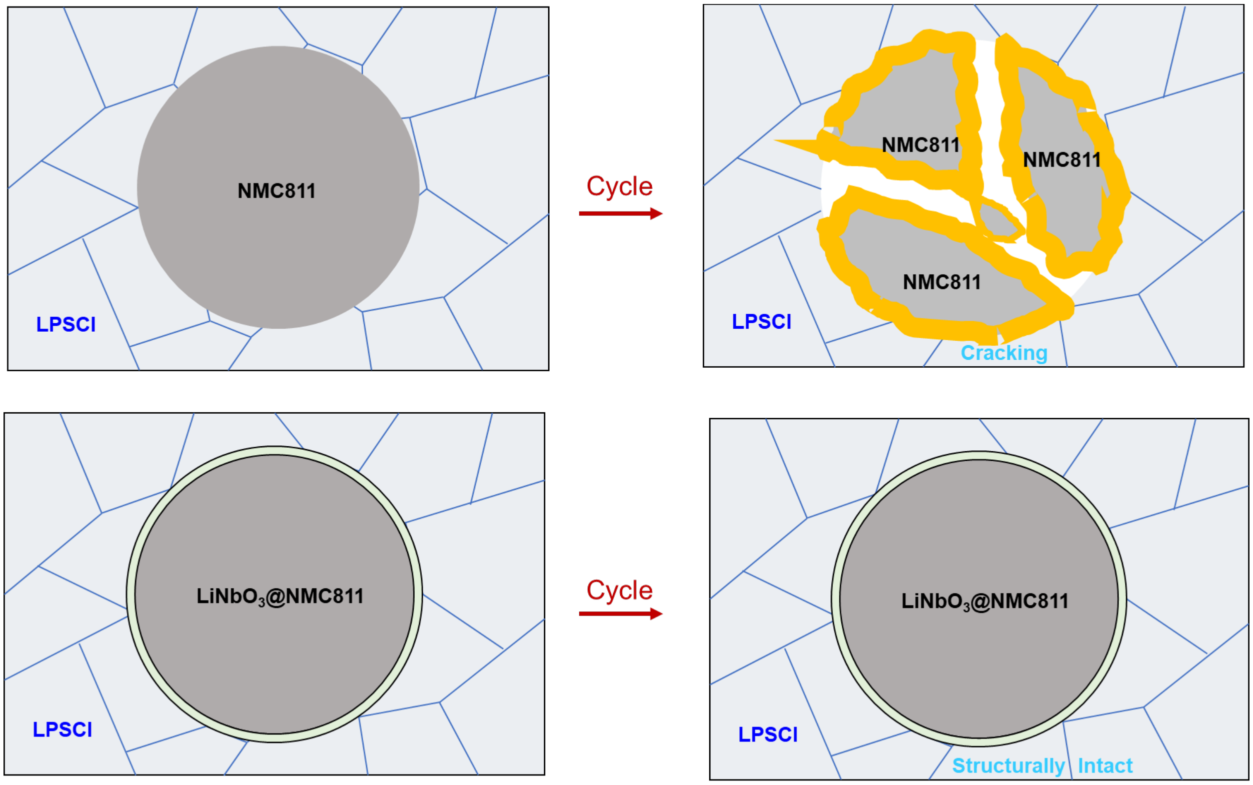

Fig. S18 Schematic diagram illustrating the role of the LNO coating in mitigating surface side reactions while enhancing the mechanical properties of NMC particles.

The diagram also shows how the LNO coating reduces the formation of isolated domains, thereby improving the overall electrochemical performance and structural stability of the cathode material.
